# Case report: Light-chain amyloidosis responsive to selinexor in combination with daratumumab and dexamethasone (SDd) therapy

**DOI:** 10.3389/fmed.2024.1363805

**Published:** 2024-05-02

**Authors:** Xiaolu Long, Ning An, Chunhui Li, Hui Zhu, Haojie Li, Qiuxia Yu, Yimei Que, Menglei Xu, Zhe Li, Wei Chen, Shuai Wang, Di Wang, Chunrui Li

**Affiliations:** ^1^Department of Hematology, Tongji Hospital, Tongji Medical College, Huazhong University of Science and Technology, Wuhan, Hubei, China; ^2^Immunotherapy Research Center for Hematologic Diseases of Hubei Province, Wuhan, Hubei, China

**Keywords:** light-chain amyloidosis, selinexor, daratumumab, organ response, SDd therapy

## Abstract

The outcome of AL amyloidosis remains poor, particularly in patients with advanced organ involvement which takes long time to recovery. We conducted an observational study of two patients with AL amyloidosis treated with SDd regimen. Both patients successfully achieved significant hematological and organ responses without severe adverse events, and the time to organ response was remarkably shorter than previously reported. Notably, an over 15% reduction in interventricular septal thickness (IVST) was observed in patient#2 within 6 months. Up to now, SDd therapy has not been previously reported in AL amyloidosis and may be a promising option for these patients.

## Introduction

This study presents a unique and promising approach in the treatment of AL amyloidosis, a rare plasma cell disorder characterized by the deposition of amyloid fibrils in various organs. The combination therapy of selinexor, daratumumab, and dexamethasone (SDd regimen) demonstrated exceptional clinical outcomes in both relapsed/refractory and newly diagnosed patients. Notably, this case report highlights several distinctive aspects that set it apart from previous literature. Firstly, the patients exhibited rapid and significant organ responses, including hematologic, cardiac, and renal improvements. Moreover, the time to achieve organ response was remarkably shorter compared to previous studies. Additionally, the synergistic effects of selinexor and daratumumab targeting malignant plasma cells through different mechanisms may contribute to the efficacy of this therapy. Overall, these findings underscore the unique potential of SDd therapy and warrant further investigation to establish its safety and efficacy in a larger AL amyloidosis patient population.

## Case report

### Case 1

#### A case of a relapsed light-chain AL with the treatment of SDd

A 67-year-old woman who had prior treatment of VD and VCD for her ligh-chain amyloidoisis (AL) and later underwent hemodialysis was referred to our hospital for further treatment in December 2021, with symptoms including dyspnea, facial oedema, small pericardial effusion, abdominal distension and oliguria.

This patient was initially diagnosed with light-chain amyloidosis (AL) with renal involvement in October 2020 at a regional hospital based on the following laboratory findings: proteinuria of 4.3 g/24 h; serum and urine immunofixation electrophoresis revealed presence of λ type monoclonal proteins; a renal biopsy was showed substantial amyloid deposition with positive green birefringence under polarized light on Congo red staining (Supplementary Figure S1). Bone marrow biopsy and fluorescence *in situ* hybridization (FISH) both showed normal findings, with no clonal plasma cells (< 1%) and no abnormal cytogenetics.

After four cycles of first-line treatment of bortezomib and dexamethasone (VD), no hematological response [difference between the involved and uninvolved free light chain (dFLC) from 280 to 67 mg/L] was obtained, and 24-hour proteinuria increased to 5.2 g. Subsequently, VCD (bortezomib, cyclophosphamide, and dexamethasone) was selected as the treatment option, however the treatment was interrupted after two cycles due to intolerability (Table 1).

**Table 1:** Baseline characteristics of 2 patients.

	**Patient #1**	**Patient #2**
Gender	Female	Male
Age (years)	66	55
ECOG performance status	3	3
AL isotype	Lambda	Lambda
Cytogenetic at baseline	Normal	Gain (1q21) RB1 deletion IgH translocation (High risk)
Lactate dehydrogenase/(U·L-1)	57	52
Ejection fraction (%)	72	59
Interventricular septum (mm)	16	20
Organ involvement	Heart and kidney	Heart and kidney
Cardiac stage at baseline	II	III
Renal stage at baseline	II	I
Additional prior therapies	VD → VCD	No prior therapy

VCD, bortezomib, cyclophosphamide, and dexamethasone; VD, bortezomib, dexamethasone.

At admission to our hospital, her baseline serum free light chain (λFLC) was 167.8 mg/L with dFLC of 152.6 mg/L. The N-terminl pro-brain natriuretic peptide (NT-proBNP) was 4,005 pg/mL (normal range: < 125 pg/mL), and the high-sensitivity cardiac troponin T (hs-cTnT) was 26.2 ng/L. Echocardiography after admission showed the following findings: left ventricular ejection fractions (LVEF), 60%; interventricular septal thickness, 10.1 mm. The 24-h proteinuria was elevated at 15.1 g.

**Figure 1:**
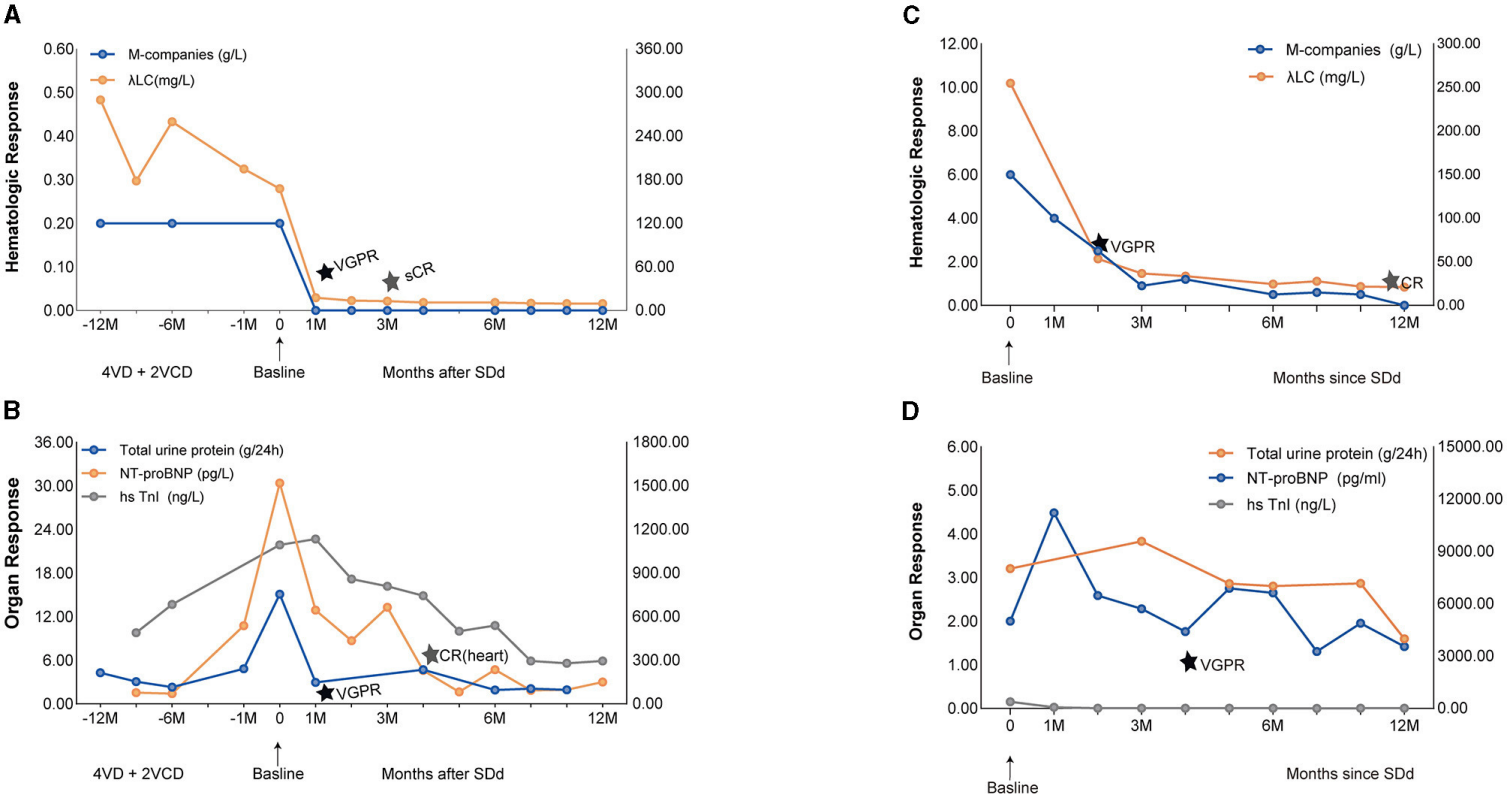
Hematologic and Organ Response of case 1 and case 2 with SDd. **(A)** Graphical illustration of M-companies and lambda free light chains during the treatment course of case 1. **(B)** Illustration of 24-h urine collection, NT-proBNP and hsTnl over time since diagnosis of AL, representative of organ response of case 1. **(C)** Graphical illustration of M-companies and lambda free light chains during the treatment course of case 2. **(D)** Illustration of 24-h urine collection, NT-proBNP and hsTnl over time since diagnosis of AL, representative of organ response of case 2.

We treated this patient with an SDd regimen (selinexor, daratumumab, dexamethasone), consisting of selinexor 60 mg orally and dexamethasone 20 mg administered weekly, accompanied by daratumumab 800 mg intravenously once a week for 4 weeks, and subsequently every other week for two doses, followed by monthly administration. After the first cycle, peripheral edema was significantly reduced; hemodialysis was no longer required; λFLC normalized (Figures 1A, B), and a hematological very good partial response (VGPR) was achieved (dFLC decreased from 152.6 to 19.5 mg/L); a renal response (>60% decrease in total 24-h urine protein) was also observed and assessed as VGPR. A hematological stringent complete response (sCR) was observed in the third cycle (λFLC 13.7 mg/L, dFLC 7.2 mg/L). The patient achieved a cardiac response of VGPR after the second cycle (>60% decrease in NT-proBNP) and CR in the fourth cycle (NT-proBNP returned to normal). The patient has completed 12 months of treatment and is currently still on SDd treatment without any signs of relapse, with λFLC in the normal range and stable cardiac function. The hematological response remains in stringent complete response (sCR) to date (Table 2).

**Table 2:** Summary of hematologic and organ responses.

**Laboratory assessments**	**Patient #1**					**Patient #2**				
	**Baseline**	**1 month**	**3 months**	**6 months**	**12 months**	**Baseline**	**1 month**	**3 months**	**6 months**	**12 months**
Urine protein (mg/24 hr)	15.094	3.1	—	1.9	—	3.2	—	—	2.8	1.6
Serum creatinine (mg/dL)	1.34	1.58	1.13	1.27	1.41	1.24	1.05	1.0	1.15	1.11
eGFR (mL/min/1.73 m^2^)	40.3	33.8	50.7	44.2	38.4	65.2	79.9	85.4	71	74.5
**Renal response**	**—**	**VGPR**	**VGPR**	**VGPR**	**VGPR**	**—**	**SD**	**SD**	**SD**	**SD**
M-component (g/L)	0.2	0	0	0	0	6	—	0.9	0.5	0
λLC (mg/L)	167.8	17.6	13	10	9.6	254.8	—	36.6	24.4	21.1
dFLC (mg/L)	152.6	10.1	5.2	9.1	2.9	236.2	—	22.2	13.1	9.6
Serum IFx	(–)	(+)	(+)	(+)	(+)	(–)	(–)	(–)	(+)	(+)
**Hematologic response**	**—**	**VGPR**	**sCR**	**sCR**	**sCR**	**—**	**PR**	**VGPR**	**VGPR**	**CR**
NT-proBNP (pg/ml)	1,520	646	665	236	151	5,011	11,219	5,711	6,633	3,549
hs-cTnI (ng/ml)	22	22.7	16.2	10.8	5.9	389.7	65.6	20.2	12.5	15
Ejection Fraction (%)	60	—	—	72	73	59	—	—	72	72
IVST (mm)	12	—	—	12	13	20	—	—	16	17
**Cardic response**	**—**	**VGPR**	**VGPR**	**CR**	**CR**	**—**	**SD**	**PR**	**VGPR**	**VGPR**
NA+(mmol/L)	141	140.5	136.3	140.6	141.6	131.4	135.4	138.1	138.3	138.6
Hemoglobin (g/dL)	86	72	76	87	93	130	140	101	108	105
ANC (/L)	3.4 × 10^9^/L	2.56 × 10^9^/L	3.64 × 10^9^/L	2.31 × 10^9^/L	2.43 × 10^9^/L	5.63 × 10^9^/L	3.45 × 10^9^/L	5.32 × 10^9^/L	4.9 × 10^9^/L	4.38 × 10^9^/L
Platelet count (/L)	207 × 10^9^/L	126 × 10^9^/L	132 × 10^9^/L	115 × 10^9^/L	142 × 10^9^/L	271 × 10^9^/L	230 × 10^9^/L	290 × 10^9^/L	232 × 10^9^/L	196 × 10^9^/L

ANC, Absolute Neutrophil Count; dFLC, difference between involved and uninvolved free light chain; eGFR, estimated glomerular filtration rate; HB, hemoglobin; hs-cTnI, elevated highly sensitive troponin I; IFx, immunofixation; IVST, interventricular septum thickness; NA+, sodium; NT-proBNP, N-terminal pro-B-type natriuretic peptide; PLT, platelets; λLC, lambda free light chain.

During the first cycle of therapy, the patient experienced a grade 3 anemia (hemoglobin 77 g/L). However, no neutropenia or thrombocytopenia of any grade was reported. The patient also experienced a grade 2 dyspepsia, vomiting, and early satiety, which led to a dose deduction of selinexor to 40 mg once weekly starting on cycle 3. These side effects also occurred in the third and fourth cycles, and grade 1 weight loss was reported during cycle 5, prompting further dose reduction of selinexor to 20 mg once a week (Table 3).

**Table 3:** Most common all-grade TEAEs of two patients.

**Classification of adverse toxicity reactions**		**Case 1 (grade)**	**Case 2 (grade)**
Hematological toxicity	Thrombocytopenia	1	0
	Neutropenia	0	0
	Anemia	3	2
Non-hematological toxicity	Nausea	2	2
	Vomiting	2	2
	Anorexia	2	2
	Fatigue	2	2
	Blurred vision (eye disorders)	1	0
	Weight loss	1	1
	Hyponatremia	0	1

TEAE, treatment-emergent adverse event.

We also assessed the patient's quality of life before and after SDd treatment using the EORTC QLQ-C30 (version 3). As a result of the treatment, the patient's role function and physical function showed significant improvements. Symptoms such as fatigue, nausea and vomiting, sleep disturbances, and loss of appetite were alleviated compared to the pre-treatment situation.

### Case 2

#### A case of SDd therapy in a newly diagnosed light-chain AL

In January 2022, a 54-year-old man was referred to our hospital for his chest tightness and dizziness. Prior to this, he was diagnosed with coronary atherosclerotic heart disease through coronary angiography at another hospital. Despite receiving secondary preventative therapy, the patient's symptoms did not significantly improve after 2 months.

The patient continued to suffer from symptoms such as fatigue, shortness of breath during mild to moderate physical activity, weight loss, and bilateral lower extremity edema. Upon admission, the patient presented with dyspnea and hypotension, with a blood pressure of 70/50 mmHg, and had an ECOG performance status score of 3. Laboratory investigations revealed λFLC of 254.8 mg/L, dFLC of 236.2 mg/L, and a κ/λ ratio of 38.4. The M-component showed an immunoglobulin (IgG)-λ of 6 g/L. Bone marrow biopsy demonstrated 5% of clonal plasma cells restricted for λ, and abnormal cytogenetics on FISH showed a gain of 1q21 (CKS1B), RB1 deletion, and IgH translocation. Echocardiography revealed asymmetric hypertrophic cardiomyopathy with an interventricular septal thickness of 2 cm and an ejection fraction (EF) of 59%. Cardiac magnetic resonance imaging (MRI) showed severe concentric hypertrophy with diffuse left ventricular myocardial delayed hyperenhancement consistent with cardiac amyloidosis (Supplementary Figure S2). Cardiac biomarkers were markedly abnormal, with NT-proBNP at 3,178 pg/mL and hs-cTnI at 389.7 ng/L. Single-photon emission computed tomography (SPECT) fusion imaging ruled out transthyretin-related cardiac amyloidosis (ATTR-CA). Additionally, the patient showed signs of renal impairment with proteinuria at 3.2 g/24 h. Based on these findings, the patient was diagnosed with AL with involvement of the heart and kidneys (Table 1).

We treated this patient with SDd regimen (selinexor, daratumumab, dexamethasone) at this time. A very good hematological partial response (VGPR) was achieved after two cycles of treatment, with dFLC decreased from 236.2 to 39.4 mg/L (Figures 1C, D). After cycle 6 the absolute value of the involved FLC normalized to 24.4 mg/L, and the IgG-λ was undetectable. A complete hematological response (CR) was observed at 12 month. With regard to cardiac response, there was a greater than 60% decrease in NT-proBNP (from 11,219 pg/mL to 4,461 pg/mL), which was achieved at the fourth cycle of therapy and assessed as VGPR. Six months later, the patient's left ventricular ejection fraction (LVEF) had recovered to 72% and the hypertrophy of the ventricular septum had reversed (from 2 cm to 1.6 cm). A renal response of PR was achieved after cycle 12, with a ≥30% decrease in total 24-h urine protein. After SDd treatment, the patient's clinical condition significantly improved, with no symptoms of hypotension, improved exercise tolerance, and a notable improvement in functional status. The patient gained 10 kg of body weight, and his performance status improved to ECOG score of 1 (Table 2).

During the second cycle of treatment, the patient experienced grade 1 hyponatremia, which subsequently improved. No grade 3 or worse hematologic adverse events were observed after three cycles of treatment. However, during the first six cycles, the patient reported nausea and loss of appetite. As a result, the dose of selinexor was reduced to 40 mg once weekly, and then to 20 mg at cycle 7, which effectively alleviated gastrointestinal adverse events (Table 3).

After comparing the scales before and after treatment using the EORTC QLQ-C30 (version 3), we observed a slight deterioration in symptoms such as fatigue, nausea, vomiting, and pain. However, the patient's physical function, emotional function, cognitive function, social function, and overall quality of life showed significant improvement. The patient is now able to perform activities of daily living and locomotion with ease, which demonstrates the overall benefit of the treatment.

## Discussion

Light chain amyloidosis (AL) is a disease caused by the misfolding of monoclonal immunoglobulin light chains leading to their deposition as amyloid in various tissues and organs, resulting in organ dysfunction and failure. This condition is predominantly associated with the abnormal proliferation of clonal plasma cells, wherein affected organs display a typical distribution (1, 2). The clinical presentation and prognosis of AL amyloidosis depend on the pattern and severity of organ involvement. Unfortunately, the prognosis for individuals with advanced organ involvement remains bleak, with a median survival time of only 4 to 6 months. Heart failure and nephrotic syndrome are commonly observed in AL amyloidosis, and the heart and kidney are often the first organs to be affected (3, 4). In this study, we conducted an observational study of two patients with AL amyloidosis and collected data from them using the SDd regimen in clinical practice to evaluate the efficacy and safety of this treatment protocol and provide additional information for clinical applications.

Most current strategies for AL amyloidosis are targeted therapies against clonal plasma cells. Reducing the circulating amyloidogenic LCs through suppression of the plasma cell clone leads to extended survival and improved organ function (5). Recovery of organ function typically lags behind the hematological response and may take months to years (6). Chemotherapy combinations are primarily derived from multiple myeloma, with some modifications to account for amyloid-related organ damage. The use of drugs such as bortezomib, daratumumab, lenalidomide, melphalan and autologous hematopoietic stem cell transplantation has induced hematological and organ responses, and prolonged survival in patients with AL amyloidosis. The median time of traditional treatment strategie (proteasome inhibitors) to at least a VGPR was 3 cycles of therapy. Crucially, the organ function improvement is slow and limited (20% patients at 6–12 months) (7–11).

Daratumumab is the first fully human monoclonal antibody to target CD38 and was approved and marketed in China in 2019 for the treatment of adult patients with relapsed and refractory multiple myeloma (RR/MM). CD38 is a type II transmembrane glycoprotein expressed on the plasma cells (PCs), making it a potential target for monoclonal antibody therapy (12). Daratumumab induces PC death via cell-mediated cytotoxicity, antibody-dependent phagocytosis, complement-dependent cytotoxicity, and apoptosis (13). The efficacy of daratumumab-based therapy, both as monotherapy and in combination in the treatment of RR AL amyloidosis has been demonstrated in several retrospective and prospective studies, with an overall response rate (ORR) over 80% (14). Nine studies reported the use of daratumumab as a monotherapy (DMT), i.e., daratumumab alone or in combination with dexamethasone. The hematologic ORR observed ranged from 55% to 100%, the CR was 0% to 43%, the cardiac response rate was seen in 22% to 67% of the patients in the DMT groups, while the renal response was observed in 18% to 80% (15). Chung et al. retrospectively evaluated 72 patients with previously treated AL amyloidosis who received daratumumab monotherapy with dexamethasone. Results showed that 77% of patients achieved a hematologic response (defined as PR+VGPR+CR), with a median time-to-hematologic response of 1 month. Among evaluable patients, 55% achieved a cardiac response, with a median response time of 3.2 months amongst responders, and 52% achieved a renal response, with a median response time of 6 months amongst responders (16). Recovery of organ function typically takes much longer than the hematological response. Daratumumab is the most effective medication presently available for the induction of remission in AL, and in our cases, patients on the SDd regime, whose time to achieve remission was even shorter.

Selinexor is a medication that functions as a first-in-class oral selective inhibitor of exportin 1 (XPO1). XPO1 inhibition impacts tumor cells through three core mechanisms: increasing nuclear levels and activation of tumor suppressor proteins (TSPs), trapping oncoprotein mRNA in the nucleus to reduce oncoprotein levels, and retaining activated glucocorticoid receptor (GR) in the nucleus (17). Additionally, XPO1 exports immune response regulators such as IkB, the inhibitor of the transcription factor nuclear factor-kB (NF-kB), leading to dysregulated cellular growth signaling and an anti-apoptotic state (18). Notably, Selinexor has also been shown to disrupt the 3D nuclear organization of telomeres in cancer cells vs. normal cells, resulting in antitumor activity, and to resensitize hypoxia-induced bortezomib-resistant clonal plasma cells to bortezomib, thereby exhibiting antitumor activity (19). This medication is capable of inducing apoptosis of tumor cells by increasing the nuclear retention of tumor suppressor proteins, glucocorticoid receptor, and mRNA of oncogenic proteins. In 2019, the FDA approved selinexor for the treatment of refractory or relapsed multiple myeloma (RR/MM) (20). Clinical studies have demonstrated that a regimen of selinexor in combination with dexamethasone is effective and safe in patients with MM who have received prior lines of therapy. Selinexor and dexamethasone are also being tested in combination with daratumumab (SDd) in the STOMP trial. Results show a 73% overall response rate (ORR) in the 30 daratumumab-naive patients with PI/IMiD refractory MM, which is more promising than the daratumumab monotherapy which has ORR of ~29% and selinexor monotherapy which has ORR of ~21% in patients with quad refractory MM (21). However, there are only limited studies on the use of selinexor in AL amyloidosis. David M. Hughes reported the first known case where selinexor in combination with dexamethasone (Sd) was used in a patient with relapsed AL amyloidosis after failing from VCD and daratumumab therapies. The patient experienced a 38% decrease from baseline in dFLC and achieved a renal response of 55% decrease in 24-h urinary protein after 3 cycles of Sd, indicating that low-dose selinexor in combination with dexamethasone was effective in a relapsed AL amyloidosis patient (22). However, to the best of our knowledge, the efficacy and safety of SDd therapy for AL has not been reported previously in the literature.

In this study, two enrolled patients met the following inclusion criteria: 1. Patients aged ≥18 years, diagnosed with systemic light-chain amyloidosis. 2. Patients receiving or planning to receive the SDd regimen as part of their treatment. 3. Routine hematology tests must meet specific requirements upon commencement of the regimen, including ANC ≥ 1.0 × 10^9^/L, RBC ≥ 8.0 g/dL, and PLT ≥ 75 × 10^9^/L. 4. Measurable disease of light-chain amyloidosis was defined by at least one of the following: a serum monoclonal (M)-protein ≥ 0.5 g/dL by protein electrophoresis, or serum free light chains ≥ 50 mg/L with an abnormal kappa:lambda ratio or dFLC ≥ 50 mg/L. 5. Patients were required to be willing to provide personal information, details of their medical history, and cooperate with clinical diagnosis and treatment management. 6. Understanding and signing the Informed Consent Form (ICF) was essential.

The study was based on the Mayo 2012 staging system (23) for AL amyloidosis, with the following indicators: 1. NT-proBNP > 1,800 ng/L; 2. cTnT > 0.025 ug/L; 3. dFLC > 180 mg/L. Stage I: all indicators are below the threshold; Stage II: 1 indicator is above the threshold; Stage III: 2 indicators are above the threshold; Stage IV: all 3 indicators are above the threshold.

Patient #1 was diagnosed with relapsed refractory AL. After receiving the SDd regimen, the patient achieved a very good partial hematologic response (VGPR), as well as cardiac and renal responses in the first month, and remained in stringent complete response (sCR) 12 months after completing the therapy. This significant improvement or recovery of organ function in the early phase, which is rarely reported in this setting of patients, was observed, and the time to achieve organ response was remarkably shorter than previously reported by other authors. Patient #2 was newly diagnosed with AL and achieved a hematologic VGPR after two cycles of therapy. The M-component showed an overall downward trend, although serum immunofixation remained positive for 9 months, likely due to therapeutic antibodies such as daratumumab interfering with the measurement of circulating M-protein. This patient also achieved a cardiac response with VGPR after six cycles, and surprisingly, interventricular septal thickness (IVST) in patient 2 was also significantly reduced (-15%). During follow-up, a decline in proteinuria and an improvement in renal function were observed.

The main adverse event of Daratumumab is infusion-related reactions, with the majority being grade 1/2 reactions (24). Long-term adverse reactions are mainly caused by Selinexor. Early monitoring and prompt adjustment of seliniexor dose can help reduce adverse events, the doses of Selinexor for two patients in our study were reduced. The most common non-hematologic treatment-related adverse events are nausea, vomiting, fatigue, and asthenia (25). Selinexor-induced vomiting generally occurs 24–48 h after administration, and therefore, aprepitant was used to prevent delayed emesis in our patients. Additionally, nausea and vomiting were not exceed grade 2. Fatigue/asthenia is also a common symptom after Selinexor administration, ranging from grade 2 to 3 for the two patients; however, these symptoms gradually decrease or disappear as treatment is completed.

Selinexor is a selective inhibitor of exportin 1 (XPO1), which can suppress the proliferation of monoclonal plasma cells and induce their apoptosis by promoting the nuclear retention of tumor suppressor proteins, mRNAs of oncogenic proteins, and glucocorticoid receptors. Daratumumab, as a CD38-directed monoclonal antibody, targets malignant monoclonal plasma cells and depletes them. Both Selinexor and Daratumumab have superior killing effects on abnormal plasma cells, relying on different mechanisms, which may result in synergistic and complementary effects between them. Therefore, selinexor in combination with daratumumab and dexamethasone (SDd) can produce a marked synergistic effect from a mechanistic point of view and present notable potential applications against AL amyloidosis in terms of etiological and pathological perspective. The current study demonstrated that SDd therapy in both replased/refractory and newly diagnosed AL patients can achieve significant organ response, with the time to organ response being remarkably shorter than previously reported. Further trials are warrant to determine the safety and efficacy of novel SDd therapy in AL patient population.

## Data availability statement

The original contributions presented in the study are included in the article/Supplementary material, further inquiries can be directed to the corresponding authors.

## Ethics statement

The studies involving humans were approved by Tongji Hospital, Tongji Medical College, Huazhong University of Science and Technology. The studies were conducted in accordance with the local legislation and institutional requirements. Written informed consent for participation was not required from the participants or the participants' legal guardians/next of kin in accordance with the national legislation and institutional requirements. Written informed consent was obtained from the individual(s) for the publication of any potentially identifiable images or data included in this article.

## Author contributions

XL: Writing – original draft, Writing – review & editing. NA: Visualization, Writing – original draft. ChunhL: Methodology, Writing – original draft, Writing – review & editing. HZ: Writing – review & editing. HL: Writing – review & editing. QY: Writing – review & editing. YQ: Writing – review & editing. MX: Writing – original draft, Writing – review & editing. ZL: Formal analysis, Writing – review & editing. WC: Writing – review & editing. SW: Writing – review & editing. DW: Conceptualization, Supervision, Writing – review & editing. ChunrL: Conceptualization, Supervision, Writing – review & editing.

## References

[1] Zanwar S Gertz MA Muchtar E. Immunoglobulin light chain amyloidosis: diagnosis and risk assessment. J Natl Compr Canc Netw. (2023) 21:83–90. 10.6004/jnccn.2022.7077 36630897 PMC10164359

[2] Oberti L Rognoni P Barbiroli A Lavatelli F Russo R Maritan M. Concurrent structural and biophysical traits link with immunoglobulin light chains amyloid propensity. Sci Rep. (2017) 7:16809. 10.1038/s41598-017-16953-7 29196671 PMC5711917

[3] Kumar SK Hayman SR Buadi FK Roy V Lacy MQ Gertz MA. Lenalidomide, cyclophosphamide, and dexamethasone (CRd) for light-chain amyloidosis: long-term results from a phase 2 trial. Blood. (2012) 119:4860–7. 10.1182/blood-2012-01-407791 22504925 PMC3418771

[4] Staron A Zheng L Doros G Connors LH Mendelson LM Joshi T. Marked progress in AL amyloidosis survival: a 40-year longitudinal natural history study. Blood Cancer J. (2021) 11:139. 10.1038/s41408-021-00529-w 34349108 PMC8338947

[5] Varga C Titus SE Toskic D Comenzo RL. Use of novel therapies in the treatment of light chain amyloidosis. Blood Rev. (2019) 37:100581. 10.1016/j.blre.2019.05.005 31167719

[6] Diomede L Rognoni P Lavatelli F Romeo M Del FE Cantu L. A Caenorhabditis elegans-based assay recognizes immunoglobulin light chains causing heart amyloidosis. Blood. (2014) 123:3543–52. 10.1182/blood-2013-10-525634 24665135 PMC4047494

[7] Wechalekar AD Lachmann HJ Offer M Hawkins PN Gillmore JD. Efficacy of bortezomib in systemic AL amyloidosis with relapsed/refractory clonal disease. Haematologica. (2008) 93:295–8. 10.3324/haematol.11627 18245653

[8] Roussel M Merlini G Chevret S Arnulf B Stoppa AM Perrot A. A prospective phase 2 trial of daratumumab in patients with previously treated systemic light-chain amyloidosis. Blood. (2020) 135:1531–40. 10.1182/blood.2019004369 32108228

[9] Dispenzieri A Lacy MQ Zeldenrust SR Hayman SR Kumar SK Geyer SM. The activity of lenalidomide with or without dexamethasone in patients with primary systemic amyloidosis. Blood. (2007) 109:465–70. 10.1182/blood-2006-07-032987 17008538

[10] Palladini G Milani P Foli A Obici L Lavatelli F Nuvolone M. Oral melphalan and dexamethasone grants extended survival with minimal toxicity in AL amyloidosis: long-term results of a risk-adapted approach. Haematologica. (2014) 99:743–50. 10.3324/haematol.2013.095463 24213149 PMC3971085

[11] Sanchorawala V Sun F Quillen K Sloan JM Berk JL Seldin DC. Long-term outcome of patients with AL amyloidosis treated with high-dose melphalan and stem cell transplantation: 20-year experience. Blood. (2015) 126:2345–7. 10.1182/blood-2015-08-662726 26443620

[12] de Weers M Tai YT van der Veer MS Bakker JM Vink T Jacobs DC. Daratumumab, a novel therapeutic human CD38 monoclonal antibody, induces killing of multiple myeloma and other hematological tumors. J Immunol. (2011) 186:1840–8. 10.4049/jimmunol.1003032 21187443

[13] Sanchez L Wang Y Siegel DS Wang ML. Daratumumab: a first-in-class CD38 monoclonal antibody for the treatment of multiple myeloma. J Hematol Oncol. (2016) 9:51. 10.1186/s13045-016-0283-0 27363983 PMC4929758

[14] Palladini G Perfetti V Perlini S Obici L Lavatelli F Caccialanza R. The combination of thalidomide and intermediate-dose dexamethasone is an effective but toxic treatment for patients with primary amyloidosis (AL). Blood. (2005) 105:2949–51. 10.1182/blood-2004-08-3231 15572585

[15] Shragai T Gatt M Lavie N Vaxman I Tadmor T Rouvio O. Daratumumab for relapsed AL amyloidosis-When cumulative real-world data precedes clinical trials: a multisite study and systematic literature review. Eur J Haematol. (2021) 106:184–95. 10.1111/ejh.13535 33090552

[16] Chung A Kaufman GP Sidana S Eckhert E Schrier SL Lafayette RA. Organ responses with daratumumab therapy in previously treated AL amyloidosis. Blood Adv. (2020) 4:458–66. 10.1182/bloodadvances.2019000776 32027745 PMC7013253

[17] Gandhi UH Senapedis W Baloglu E Unger TJ Chari A Vogl D. Clinical implications of targeting XPO1-mediated nuclear export in multiple myeloma. Clin Lymphoma Myeloma Leuk. (2018) 18:335–45. 10.1016/j.clml.2018.03.003 29610030

[18] Taylor-Kashton C Lichtensztejn D Baloglu E Senapedis W Shacham S Kauffman MG. XPO1 inhibition preferentially disrupts the 3D nuclear organization of telomeres in tumor cells. J Cell Physiol. (2016) 231:2711–9. 10.1002/jcp.25378 26991404 PMC5111786

[19] Sun Q Chen X Zhou Q Burstein E Yang S Jia D. Inhibiting cancer cell hallmark features through nuclear export inhibition. Signal Transduct Target Ther. (2016) 1:16010. 10.1038/sigtrans.2016.10 29263896 PMC5661660

[20] Haider S Ahmad N Anaissie E Driscoll JJ. Future directions in the clinical management of amyloid light-chain amyloidosis. Leuk Lymphoma. (2014) 55:2241–51. 10.3109/10428194.2013.876630 24359238

[21] Podar K Shah J Chari A Richardson PG Jagannath S. Selinexor for the treatment of multiple myeloma. Expert Opin Pharmacother. (2020) 21:399–408. 10.1080/14656566.2019.1707184 31957504

[22] Hughes DM DeMari S Hassan H Sanchorawala V Sloan JM. Safety, tolerability, and efficacy of selinexor in a patient with relapsed light chain (AL) amyloidosis. Clin Lymphoma Myeloma Leuk. (2021) 21:e460–3. 10.1016/j.clml.2021.01.001 33716055

[23] Kumar S Dispenzieri A Lacy MQ Hayman SR Buadi FK Colby C. Revised prognostic staging system for light chain amyloidosis incorporating cardiac biomarkers and serum free light chain measurements. J Clin Oncol. (2012) 30:989–95. 10.1200/JCO.2011.38.5724 22331953 PMC3675680

[24] Zhang YH Xu F Xu CQ Zhang ZT Jiao ZJ. Effect of daratumumab combined with chemotherapy on immune function in patients with relapsed/refractory multiple myeloma and observation of its clinical efficacy. Pak J Med Sci. (2023) 39:248–52. 10.12669/pjms.39.1.6667 36694744 PMC9843002

[25] Gasparetto C Schiller GJ Tuchman SA Callander NS Baljevic M Lentzsch S. Once weekly selinexor, carfilzomib and dexamethasone in carfilzomib non-refractory multiple myeloma patients. Br J Cancer. (2022) 126:718–25. 10.1038/s41416-021-01608-2 34802051 PMC8605887

