# Supplementary material for: Case report: Light-chain amyloidosis responsive to selinexor in combination with daratumumab and dexamethasone (SDd) therapy
Source: Front Med (Lausanne). 2024 May 2;11:1363805. doi: 10.3389/fmed.2024.1363805 (PMC11096580; doi:10.3389/fmed.2024.1363805)

Supplemental files

Figure S1.

Representative images of AL of renal staining of case 1.

1. Panel A show light micrograph of a specimen stained with Congo red.
2. Panel B show the specimen in panel A under polarized light.


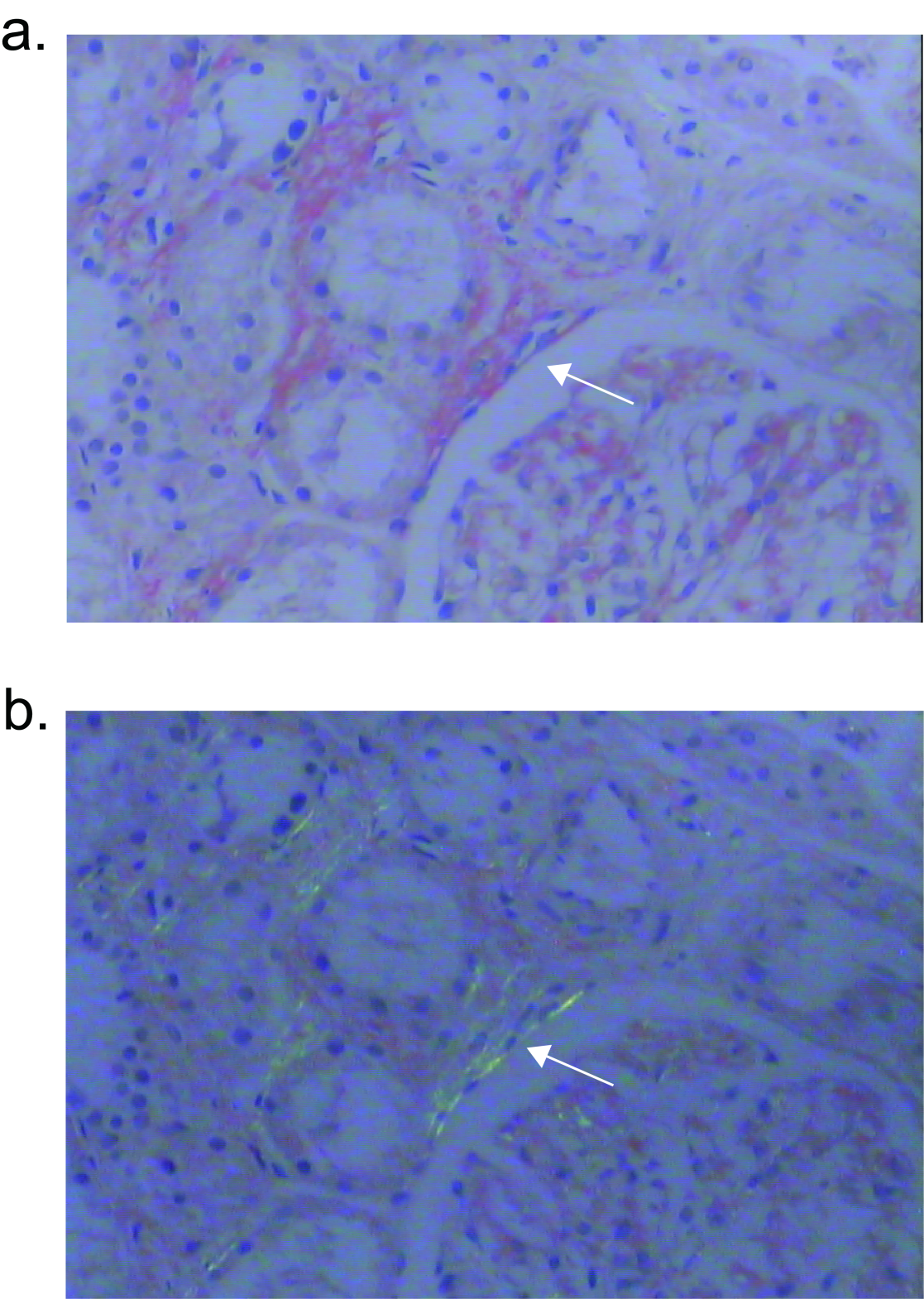


Figure S2.

Cardiac magnetic resonance imaging findings of case 2.

MRI showed severe concentric hypertrophy with diffuse left ventricular myocardial delayed hyperenhancement in a pattern consistent with cardiac amyloidosis.


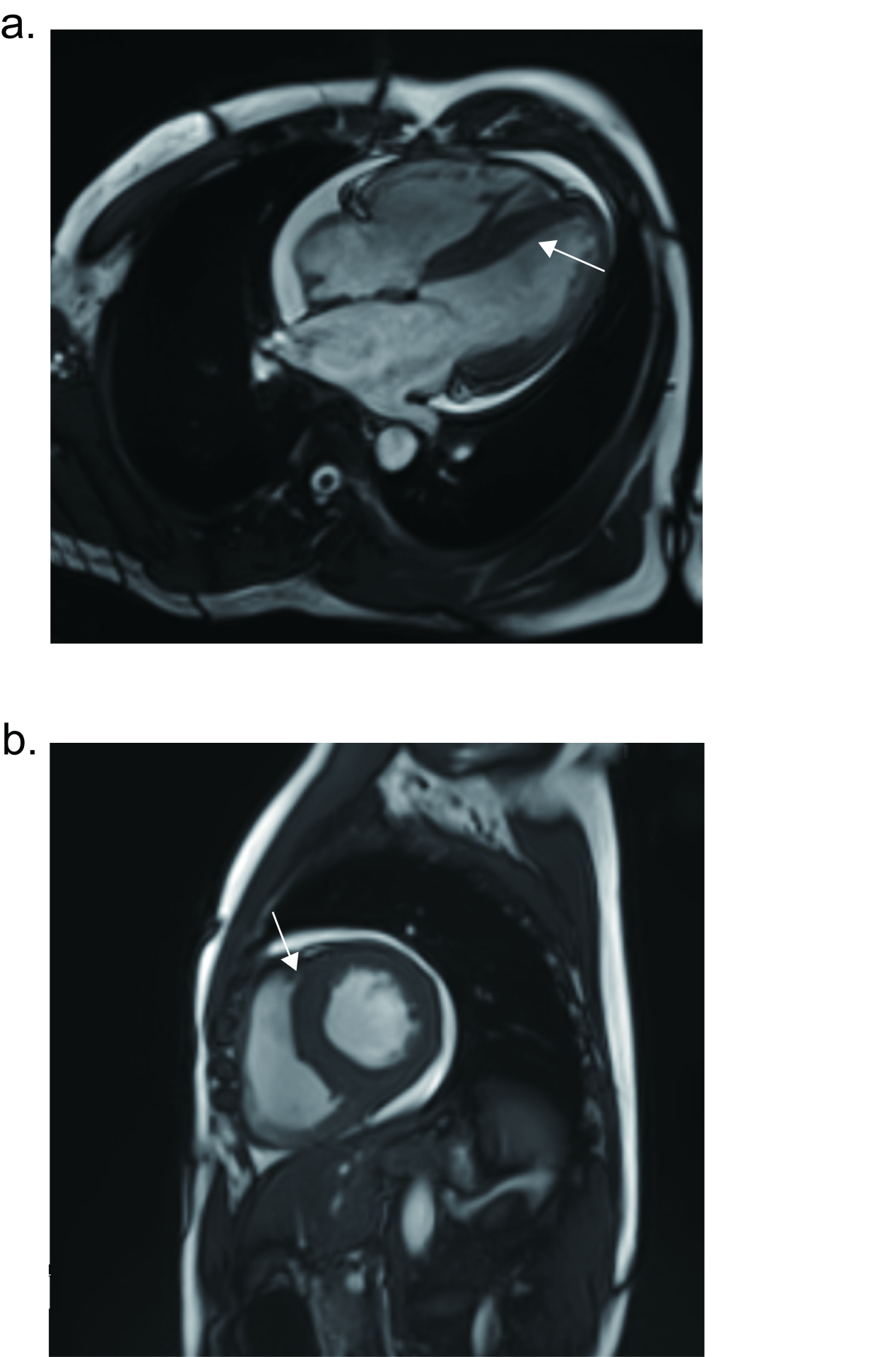

Supplement: Supplementary file 1 [file Data_Sheet_1.docx]
